# Effect of toothbrush type on biofilm and periodontal health in orthodontic patients

**DOI:** 10.6026/973206300212171

**Published:** 2025-07-31

**Authors:** Perthish Sharma, Rohit Sharma, Vidyesh Nadkerny, Harleen Kaur Sohi, Bharathi V.S., Amanda Antoinette Rebello, Miral Mehta

**Affiliations:** 1 Department of Orthodontics and Dentofacial Orthopaedics, Sri Guru Ram Das Institute of Dental Sciences and Research, Amritsar, Punjab, India; 2 Department of Dentistry, Amaltas Institute of Medical Sciences, Dewas, Madhya Pradesh, India; 3 Department of Orthodontics and Dentofacial Orthopaedics, Daswani Dental College & Research Centre, Kota, Rajasthan, India; 4 General Dentist in Massachusetts, Pittsfield, Massachusetts, US; 5 Department of orthodontics, The Oxford Dental College, Bangalore, Karnataka, India; 6 Department of Dental Surgery, Kalinga Institute of Dental Sciences, KIIT (Deemed to be University), KIIT Campus, Patia Bhubaneswar, Odisha - 751024, India; 7 Department of Pediatric and Preventive Dentistry, Karnavati School of Dentistry, Karnavati University, Gandhinagar, Gujarat, India

**Keywords:** Orthodontic brackets, biofilm, periodontal health, powered toothbrush, sonic toothbrush, oral hygiene

## Abstract

The sonic brush lowered the growth of biofilm on orthodontic brackets more successfully than both powered and manual toothbrushes did. We show that the subjects in Group C (Sonic tooth brushes) achieved the most substantial progress in plaque index and gingival index scores as well as bleeding on probing results. The biofilm formation on orthodontic brackets was least prominent on metallic brackets as opposed to ceramic or composite resin brackets. People using sonic or powered toothbrushes followed their oral hygiene instructions correctly. Thus, Patients undergoing orthodontic treatment should use sonic toothbrushes for achieving their best periodontal health.

## Background:

Orthodontic dental treatment acts as a vital tool to fix patient malocclusions together with aesthetic dental improvements. Patients face substantial impediments to oral hygiene practice when using fixed orthodontic appliances because these devices create numerous surfaces that allow plaque buildup. Treatment areas affected by brackets and bands together with archwires become hard to clean properly which raises risks for negative dental outcomes and tooth enamel erosion throughout orthodontic therapy [1]. Orthodontic bracket surfaces tend to form biofilms that pose major problems for oral hygiene state as well as increased risk for dental caries and periodontal problems [2]. Professional management of the biofilm is essential since Streptococcus mutans along with other cariogenic microorganisms produce biofilm that can transform into white spot lesions and dental caries and periodontal inflammation. Orthodontic bracket materials demonstrate different degrees of biofilm attachment due to diverse materials properties. The formation of biofilm on metallic brackets made of stainless steel has been shown to be lower when compared to ceramic and composite resin-based alternatives. Alam *et al.*'s study detected that metallic brackets exhibited a mean optical density (OD) value of 0.25 ± 0.05 for biofilm formation which proved less than what ceramic brackets (0.42± 0.08) and composite resin brackets (0.58 ± 0.07) demonstrated [2]. A study conducted by the American Journal of Orthodontics and Dentofacial Orthopaedics demonstrated that electric toothbrushes promote better oral hygiene for those with braces, with the evidence being more pronounced for patients with initially poor oral hygiene [3]. A review of several studies showed an 11% reduction in plaque at one to three months for powered toothbrushes and a 21% reduction after three months of use compared to manual brushing [1]. Conversely, other research has found no significant differences between manual and powered toothbrushes in this population. A randomized clinical trial by Saruttichart *et al.* comparing manual versus powered toothbrushes in 92 adolescent patients found no significant differences in plaque index (PI), gingival index (GI), or bleeding on probing (BoP) between the two groups over a 12-month follow-up period [4, 5].

Both groups showed worsening periodontal health indicators following placement of fixed appliances, suggesting that toothbrush type alone may not be sufficient to maintain optimal oral hygiene during orthodontic treatment. The conflicting evidence extends to sonic toothbrushes as well. Some comparative studies have found sonic brushes to be superior in reducing gingivitis, plaque and interdental bleeding compared to manual orthodontic and conventional powered brushes [6, 7]. However, a systematic review and meta-analysis comparing manual and powered toothbrushes for orthodontic patients concluded that using either manual or powered tooth brushing with fixed orthodontic appliances does not significantly reduce plaque or gingival indexes at 4 weeks and 8 weeks [6]. Therefore, it is of interest to evaluate the comparative effectiveness of manual, conventional powered and sonic toothbrushes on biofilm formation and periodontal health across different orthodontic bracket materials.

## Materials and Methods:

## Study designs:

Ninety patients scheduled to commence fixed orthodontic treatment were recruited for the study. Inclusion criteria were: (1) patients aged 12-18 years; (2) requiring comprehensive fixed orthodontic treatment in both arches; (3) no history of previous orthodontic treatment; (4) absence of systemic diseases affecting periodontal health; (5) non-smokers; and (6) provision of written informed consent from patients and their guardians. Exclusion criteria included: (1) patients with severe crowding requiring extractions; (2) presence of active periodontal disease; (3) use of antibiotics or antiseptic mouthwashes in the preceding three months; and (4) physical or mental disabilities affecting manual dexterity.

## Sample size calculation:

Sample size was calculated using G*Power software (version 3.1), based on a previous similar study. With an effect size of 0.35, alpha error of 0.05 and power of 80%, a minimum of 84 participants (28 per group) was required. Accounting for a potential dropout rate of 10%, 90 participants (30 per group) were enrolled.

## Randomization and allocation:

Participants were randomly assigned to one of three groups using a computer-generated random sequence with allocation concealment ensured through opaque sealed envelopes opened only after enrollment:

[1] Group A: Manual orthodontic toothbrush

[2] Group B: Conventional powered toothbrush (oscillating-rotating)

[3] Group C: Sonic toothbrush

## Interventions:

All participants received standardized fixed orthodontic appliances with brackets of three different materials (metallic, ceramic and composite resin) distributed in different quadrants according to a predetermined pattern. Following appliance placement, participants received standardized oral hygiene instructions specific to their assigned toothbrush type. All groups were instructed to brush twice daily for two minutes using the modified Bass technique with a fluoride toothpaste (1450 ppm). Group A used a manual orthodontic toothbrush with V-shaped bristle design. Group B used a conventional powered toothbrush with oscillating-rotating action. Group C used a sonic toothbrush operating at >30,000 vibrations per minute. Participants were provided with their respective toothbrushes and instructed not to use any additional oral hygiene aids or mouthwashes during the study period.

## Outcome measures:

## Primary outcomes:

[1] Biofilm formation on brackets: Assessed using the crystal violet staining method and spectrophotometric analysis at 595 nm, expressed as optical density (OD) values. One bracket from each material type was collected from 10 randomly selected participants in each group at 12 weeks.

[2] Periodontal health parameters: Assessed at baseline (prior to appliance placement), 4 weeks, 8 weeks and 12 weeks:

1) Plaque Index (PI) using the modified Silness and Löe index

2) Gingival Index (GI) using the Löe and Silness index

3) Bleeding on Probing (BoP) as percentage of sites showing bleeding

## Secondary outcomes:

[1] Patient compliance: Assessed using a self-reported daily brushing diary

[2] Patient satisfaction: Evaluated using a visual analog scale (VAS)

## Blinding:

While participants could not be blinded to their assigned toothbrush type, the examiners conducting clinical assessments and laboratory analyses were blinded to group allocation.

## Statistical analysis:

Data analysis was performed using SPSS software (version 28.0, IBM Corp). Normality of data distribution was assessed using the Shapiro-Wilk test. Between-group comparisons were conducted using one-way ANOVA with Tukey's post-hoc test for normally distributed data and Kruskal-Wallis test with Mann-Whitney U test for non-normally distributed data. Within-group comparisons across time points were analyzed using repeated measures ANOVA with Bonferroni correction. A p-value <0.05 was considered statistically significant.

## Results:

A total of 90 participants were initially enrolled in the study, with 86 completing the 12-week follow-up (28 in Group A, 29 in Group B and 29 in Group C), representing a dropout rate of 4.4%. The demographic and baseline characteristics of participants are presented in Table 1 (see PDF). No significant differences were observed between the groups in terms of age, gender distribution, or baseline periodontal parameters, indicating successful randomization. Biofilm formation was assessed on different bracket materials at the 12-week time point. As shown in Table 2 (see PDF), significant differences were observed between toothbrush types and bracket materials. Sonic toothbrushes (Group C) demonstrated the lowest biofilm formation across all bracket materials, followed by powered toothbrushes (Group B) and manual toothbrushes (Group A) (p<0.001). Regardless of toothbrush type, metallic brackets showed significantly lower biofilm accumulation compared to ceramic and composite resin brackets (p<0.001). The interaction between toothbrush type and bracket material was statistically significant (p=0.018), indicating that the effectiveness of each toothbrush type varied by bracket material. The difference in biofilm formation between toothbrush types was most pronounced for composite resin brackets, followed by ceramic brackets and least for metallic brackets. Changes in periodontal health parameters (PI, GI and BoP) over the 12-week study period are presented in Table 3 (see PDF). All three parameters showed initial worsening after appliance placement at 4 weeks across all groups, followed by improvements at 8 and 12 weeks as participants adapted to oral hygiene regimens. At 4 weeks, no significant differences were observed between the groups for any periodontal parameter, although Group C showed a trend toward lower values. By 8 weeks, significant differences emerged, with Group C demonstrating significantly lower PI, GI and BoP compared to Groups A and B (p<0.001). This difference became more pronounced at 12 weeks, with Group C showing 47.8% lower PI, 49.4% lower GI and 53.5% lower BoP compared to Group A and 32.4% lower PI, 34.4% lower GI and 37.7% lower BoP compared to Group B (all p<l0.001). At the 12-week follow-up, the percentage of participants returning to baseline or better values for all periodontal parameters was significantly higher in Group C (72.4%) compared to Group B (51.7%) and Group A (35.7%) (p<0.001). Self-reported compliance was highest in Group C (92.3% adherence to prescribed brushing frequency), followed by Group B (86.7%) and Group A (78.2%) (p=0.013). Patient satisfaction scores were also significantly higher for Group C (mean VAS score: 8.4±1.2) compared to Group B (7.3 ± 1.4) and Group A (6.5 ± 1.6) (p<0.001).

## Discussion:

This clinical investigation tested the influence of manual orthodontic, conventional powered and sonic toothbrushes regarding biofilm development on various orthodontic bracket materials as well as periodontal health outcomes in teenage patients receiving fixed orthodontic treatment. Sonic toothbrushes proved better than conventional powered brushes and manual brushes at reducing biofilm than controlling periodontal health as patients progressed through the 12-week trial duration. The study revealed meaningful variations in biofilm development which depended on both the toothbrush type and bracket material selection. The biofilm formation remained lowest when patients used Sonic toothbrushes and these results were superior to both conventional powered brushes and manual orthodontic toothbrushes. Research by other teams has proven previously that sonic technology produces superior plaque removal results for orthodontic patients 7. Sonic toothbrushes provide effective biofilm disruption by their high-frequency vibration pattern (>30,000 strokes per minute) which generates dynamic fluid movements that exceed toothbrush bristle reach for cleaning spaces near brackets and beneath archwires. This investigation showed that metallic brackets accumulated lower biofilm volumes than both ceramic and composite resin brackets no matter which toothbrush was used. Results from Alam *et al.*'s study validate our findings showing that metallic brackets had 0.25 ± 0.05 standard optical density measurements as opposed to ceramic brackets at 0.42 ± 0.08 and composite resin brackets reaching 0.58 ± 0.07 [2]. The measured values in our research correspond to one another especially when participants used manual toothbrushes. Metallic brackets demonstrate reduced biofilm formation because their smooth surface texture and low surface energy promotes less bacterial adhesion compared to ceramic and composite resin materials that present higher surface roughness as a biofilm concession area. Different toothbrushes show varying success rates based on the type of bracket material according to the study results. Biofilm development between different toothbrush types reached its highest point when patients used composite resin brackets suggesting powered and sonic models might offer optimal results to patients with this bracket type. The placement of orthodontic appliances caused periodontal parameters (PI, GI and BoP) to worsen at week 4 in every treatment group in agreement with existing research about declining oral hygiene after appliance placement. The initial worsening of scores at week 4 can be explained by the increased plaque retention sites and limited accessibility for proper cleaning which occur due to orthodontic components. At week 8 of the experiment the sonic toothbrush group experienced superior periodontal health outcomes than either of the other groups. The results may differ because their research survey did not include sonic toothbrushes as a testing unit. The results obtained from our study match those of comparative studies because sonic brushes demonstrate better effectiveness than manual orthodontic and conventional powered brushes in eliminating gingivitis and plaque as well as interdental bleeding [7]. Hydrodynamic forces generated by sonic vibrations together with mechanical action from the bristles may contribute to superior biofilm disruption by reaching spaces that bristles alone cannot access [8, 9]. In growing individuals, the combination of anterior bite planes and fixed orthodontic appliances is an established and effective approach for correcting deep bite, achieving proper alignment, and enhancing dental and facial aesthetics [10, 11-12]. Review authors suggest the differences between this and other studies might stem from research design, participant variables or the specific powered toothbrushes included in data analysis. The sonic toothbrush group earned higher levels of both reported compliance and satisfaction compared to participants who used conventional powered or manual brushes. The enhanced compliance levels of these participants possibly resulted in improved periodontal health measurements within this group. Putting together past studies found that patients preferred powered brushes when wearing orthodontic appliances which enhanced their following of recommended brushing protocols while sonic toothbrush technology offered both new features and a superior clean [13, 14-15]. Powered toothbrushes are more effective than manual toothbrushes in reducing plaque, gingival inflammation, and biofilm accumulation in orthodontic patients. Clinical trials consistently show improved oral hygiene outcomes with powered brushing, supporting its recommendation for enhanced periodontal health during orthodontic treatment [16, 17]. Strengths of our study include the randomized controlled design, the consideration of different bracket materials and the comprehensive assessment of both biofilm formation and periodontal health parameters. However, several limitations should be acknowledged. First, the 12-week follow-up period may not capture long-term effects that could emerge over the course of orthodontic treatment, which typically spans 18-24 months [18-19].

## Conclusion:

Sonic toothbrushes demonstrated superior effectiveness compared to conventional powered and manual orthodontic toothbrushes in reducing biofilm formation on orthodontic brackets and improving periodontal health parameters in adolescent patients undergoing fixed orthodontic treatment. Metallic brackets accumulated significantly less biofilm than ceramic and composite resin brackets, regardless of toothbrush type, suggesting that bracket material selection is an important consideration for minimizing biofilm-related complications.
